# Emotional risk factors before in vitro fertilization among infertile couples in daily clinical practice in Sari in 2020–2022

**DOI:** 10.1186/s40359-024-01796-5

**Published:** 2024-05-29

**Authors:** Sommayeh Taghaddosi Kargar, Fatemeh Vakili, Sepideh Peivandi, Shayesteh Jahanfar, Forouzan Elyasi, Zeinab Hamzehgardeshi

**Affiliations:** 1 Nasibeh Nursing and Midwifery Faculty, Mazandaran University of Medical Science, , Sari, Iran; 2 School of Nursing and Midwifery, Shahid Beheshti University of Medical Sciences, Tehran, Iran; 3 Mazandaran University of Medical Sciences, Sari, Iran; 4 Department of Public Health and Community Medicine, Tufts University School of Medicine, Boston, MA USA; 5 Department of Psychiatry, School of Medicine, Mazandaran University of Medical Sciences, Sari, Iran; 6 Sexual and Reproductive Health Research Center, Mazandaran University of Medical Sciences, Sari, Iran

**Keywords:** Infertility, Social support, Depression, Anxiety, Hopelessness, Acceptance

## Abstract

**Background and Aim:**

The relationship between psychological factors and treatment outcomes with assisted reproductive technology has sparked considerable debate. This study aims to investigate the emotional risk factors in couples seeking infertility treatment using assisted reproductive technology in Sari, Iran, from 2020 to 2022.

**Materials and methods:**

This research is a cross-sectional study and emotional risk factors and other related factors were examined using the Persian version of the SCREENIVF demographic, social, and clinical status questionnaire, social, and clinical status questionnaire before using Assisted reproductive technology in 460 infertile couples selected from infertility treatment centers in Sari City, Iran. The samples were randomly selected using a table of random numbers. Data analysis was performed using SPSS version 22 software.

**Results:**

The mean age of the male and female participants were 31.70 ± 5.71 and 35.22 ± 5.48, respectively. The results regarding emotional risk factors and other related factors revealed that the variables of remarriage (*P* = 0.048) and exposure of spouse to emotional risk factors (*P* = 0.001), history of depression disorder (*P* = 0.007), and history of anxiety disorder (*P* = 0.009) were significantly correlated with the exposure of women to emotional risk factors. Furthermore, men’s exposure to emotional risk factors was significantly correlated with primary education (*P* = 0.026) and diploma (*P* = 0.043) levels, age (*P* = 0.006), and wife’s exposure to emotional risk factors (*P* = 0.001).

**Conclusion:**

By identifying infertile couples who are at risk of emotional risk factors, healthcare professionals can provide appropriate support and interventions to mitigate the emotional challenges associated with infertility. This proactive approach can significantly enhance couples undergoing infertility treatment’s well-being and mental health.

## Introduction

Infertility is considered a stimulant for psychological morbidity that reduces the efficacy of assisted reproductive technology (ART) [1]. According to the European Society of Human Reproduction and Embryology (ESHRE), approximately one in four infertile women and one in ten infertile men experience depression [2]. Although there is no consensus about the percentage of infertile couples, it is estimated that approximately 15% of couples worldwide, equivalent to around 190 million people, experience infertility issues [3].In Iran, the overall prevalence of infertility is reported at 13.2%, with primary infertility accounting for 5.2% and secondary infertility for 3.2% [4]. More than 50% of infertile couples seek medical assistance to solve their fertility challenges [5]. Assisted reproductive technology (ART) is the most commonly used approach for treating infertility and encompasses various techniques [5, 6] Among these techniques, intracytoplasmic sperm injection (ICSI) is the most prevalent, accounting for 46.6% of all treatment cycles, followed by frozen embryo replacement (FER) at 24.7% and in vitro fertilization (IVF) at 18.8% [7]. Most infertile couples believe that infertility causes significant stress [8]. The condition is a source of stress, and the treatment process, with its uncertain outcomes, adds physical and psychological burden for couples [9]. One study in United States of America indicated that approximately 30.8% of women and 10.9% of men during IVF treatment receive psychiatric diagnoses [10].

Anxiety disorders were reported in around one in seven infertile women and one in twenty infertile men after receiving the outcomes of a pregnancy test performed through In Vitro Fertilization/Intracytoplasmic sperm injection (IVF/ICSI) in Netherlands [11]. Mood disorders were observed in 26.2% of women and 9.2% of men, while anxiety affected 14.8% of women and 4.9% of men [10]. Among women undergoing infertility treatment in USA, the prevalence of major depressive disorder (MDD) ranged from 17 to 19.5%, while in men it was 15.3% or higher [12]. Another study in the Netherlands showed that the demanding nature of the treatment, both physically and emotionally, leads to premature treatment discontinuation in approximately 23–30% of couples [13]. Recent studies in Germany and the Netherlands have shown that psychological factors, particularly personal problems and relationship incompatibility, are the primary reasons for discontinuing ART [6, 14].

Early treatment discontinuation is associated with a 15% decrease in pregnancy rates [6], even in cases where ART results in a successful pregnancy and normal delivery, women still experience psychological distress. Despite successful treatment, individuals with a history of infertility are more prone to pregnancy-related anxiety, reduced postpartum self-confidence, parenting difficulties, and an increased risk of psychiatric disorders, including affective and psychotic disorders, during the first 90 days after childbirth [12]. It is commonly believed that infertility treatment is more time-consuming and painful for women than men, leading to a perception that the psychological impact of infertility is lower in men than in women [12]. Mills et al. (2009) demonstrated that infertile women experience high emotional distress [15]. Another study by Huppelschoten et al. (2011) also indicated that women are more vulnerable to psychological issues than their husbands, and the quality of life scores in this group of infertile women had decreased significantly [16]. Many infertile women believe that emotional distress can affect their ongoing infertility, and depression can diminish their motivation to continue treatment after unsuccessful attempts [12]. Women with psychological disorders often lack the necessary support, and their engagement in intensive treatments like IVF can pose a higher risk of emotional deterioration, particularly in cases of treatment failure [14]. This gender-related effect can be explained by societal gender expectations, where motherhood is often seen as a major life goal and a source of personal fulfillment [5]. Additionally, the stigma surrounding infertility prevents patients from openly discussing their problems, leading to a lack of social support. Unsuccessful treatment cycles can further elevate anxiety and depression levels, increasing the risk of suicide among women [16].

These psychological factors associated with infertility can influence patients’ decisions to discontinue treatment and reduce their chances of achieving pregnancy. Consequently, it is crucial to study both women and their partners as separate entities in the context of infertility [16]. Identifying patients at risk of psychological distress before initiating IVF treatment is of utmost importance [17, 18]. This identification enables infertility treatment centers to provide enhanced care and support, aiming to prevent emotional problems [17]. Screening Tool on Distress in Fertility Treatment (SCREENIVF), is the first screening tool used in infertility treatment centers, assesses five risk factors through a short, valid, reliable, and evidence-based self-administered questionnaire consisting of 34 items. These risk factors have been identified in prospective studies as indicators of emotional distress following unsuccessful IVF treatment.

Due to the adverse psychological effects caused by infertility, different aspects of the lives of infertile couples are affected physically, psychologically, and socially. It also causes emotional symptoms such as anxiety, depression, tension, sadness, worry, and mental concerns in them. Currently, the screening questionnaire for emotional risk factors before treatment with assisted reproductive methods has made it possible to help in the treatment process of these patients by identifying patients at risk of psychological factors and providing psychological and social support for couples. SCREENIVF aims to assign women at risk for emotional maladjustment before, during, and after treatment These findings highlight the potential of SCREENIVF in developing preventive treatment plans that target patients’ vulnerabilities [17]. Given the context mentioned above, the present study was conducted to investigate emotional risk factors and other related factors among infertile couples in Sari City.

## Methodology

### Type of study and participants

This cross-sectional study was conducted on 460 infertile couples referred to the infertility treatment center in Sari, Iran, from December 3, 2020, to January 19, 2022.

### Sample size

According to a study conducted by Lopes et al. in 2013, it was found that 47% of the samples, consisting of both women (52%) and men (30%), were at risk of experiencing emotional disorders before undergoing IVF/ICSI treatment. The following formula was used to calculate the sample size with an accuracy of 5% and a confidence level of 95%.

$$n\, = \,\frac{{Z_{1 - \frac{\alpha }{2}}^2p(1 - p)}}{{{d^2}}}$$

The calculated sample size was determined to be 383 women and 322 men. However, since the sampling was conducted from infertile couples (including both women and their husbands), it was essential to consider the potential low cooperation of men. Therefore, considering a 20% probability of incomplete questionnaire answers, the final sample size was set at 460 infertile couples.

### Inclusion and exclusion criteria

Inclusion criteria were couples who attended infertility treatment clinics for infertility treatment, infertile couples waiting for any of the IVF or ICSI treatments, willingness to participate in the study, ability to read and write, Iranian nationality and exclusion criteria were lack of willingness to continue participation in the research.

### Data collection tools

#### Demographic and clinical status questionnaires

This questionnaire encompasses personal and family information, fertility-related information, and clinical status information. The personal and family information section includes questions about age, education, occupation, employment status, ethnicity, economic status, place of residence, duration of marriage, and number of previous marriages. The fertility-related information section consists of questions regarding the cause and duration of infertility, duration of infertility treatment, type of infertility, current stage of treatment, number of previous treatments, willingness to continue treatment, intention to use embryo or oocyte donation or surrogacy, whether the participant has an adopted child and current stage of treatment. The information related to the clinical status section includes questions about the history of depression disorder, anxiety disorder, use of psychiatric drugs, past suicide attempts, and history of visiting a psychiatrist/psychologist [11, 16]. To ensure the comprehensiveness and validity of the questionnaire, a comprehensive review of relevant studies and questionnaires used in similar research was conducted. The content validity of the questionnaire was also assessed through multiple stages of review by the research team.

#### SCREENIVF questionnaire

This questionnaire is a valid and concise questionnaire comprising 34 items designed by Verhaak et al. in 2010 to identify infertile women at risk of psychological factors. The original version is in Dutch. The questionnaire includes five scales for emotional disorders, which are considered risk factors for increased emotional problems during fertility treatment. These scales or risk factors include anxiety, depression, perceived social support, hopelessness, and acceptance of infertility problems. The questionnaire consists of 10 items in the anxiety domain, seven items in the depression domain, five items in the social support domain, and 12 items in the understanding of infertility issues domain. The domain of understanding of infertility issues consists of two subcategories. The helplessness domain collects scores related to items 1, 4, 5, 6, 8, and 11, while the acceptability domain collects scores from items 2, 3, 7, 9, 10, and 12. For the anxiety domain, the threshold limit is set at 24 or above. In scoring the depression scale, the threshold limit is set at four or above. The threshold limit is set at 14 and above for helplessness, 11 and above for acceptability, and 15 and below for social support. Consequently, SCREENIVF generates a two-dimensional score for each of the five risk factor domains. Patients with a score below the threshold limit are assigned a score of zero, while those scoring equal to or above the threshold receive a score of one. The scoring range for SCREENIVF is from 0 to 5, where zero indicates the absence of risk and 5 indicates the presence of all five risk factors [9, 13] [10]. . SCREENIVF results in a risk profile that is offered to the patients. Based on this risk profile, further psychosocial care can be offered. SCREENIVF is a screening tool that can provide patients with knowledge of their risk description and give them feedback that they could profit from psychosocial support [19]. Studies indicated that the SCREENIVF is an admissible instrument to recognize women at risk for psychological maladjustment and that its usage in the clinic context is possible [20]. The SCREENIVF questionnaire was translated by Sorkhabi et al., and its validity and reliability were confirmed through the participation of 50 individuals. A post-test was conducted after two weeks, resulting in a Cronbach’s alpha coefficient of 0.7 and an ICC of 0.74 [21]. The qualitative content validity of the Persian version of the SCREENIVF questionnaire was determined by 14 experts in the field of reproductive health and psychiatry. They were asked to give their written opinion after carefully reading the questionnaire items. In addition, the grammar was qualitatively analyzed. SCREENIVF was revised according to experts’ opinions and feedback. The quantitative content validity of the questionnaire was evaluated by calculating the content validity ratio and the content validity index for the items. In measuring the content validity ratio (CVR), the scores of all items were equal to or greater than 0.57, so the items were recognized as “essential”. According to Lawsche table, the value of content validity ratio for 14 experts is higher than 51% as a necessary item in the tool. The Persian version of SCREENIVF was again given to a panel of experts to give their opinions on how clear, simple, and relevant each item is on a 4-point Likert scale. Then CVI was calculated using the formula. The following criteria were used for the qualitative CVI values: less than 0.75 is unacceptable, 0.7–0.78 was considered to be revision and correction of the item, and equal to or above 0.79 was considered appropriate. In addition, to ensure that the items were well designed, a content validity index was collected with direct feedback from a panel of experts in the field of reproductive health and psychiatry.

### Ethical consideration

#### Ethical approval

for the study was obtained from the Ethics Committee of Mazandaran University of Medical Sciences, with the research project code IRMAZUMS.REC.1398.706. A written consent form was signed by the participants if they were willing to participate in the study. The women and men were also assured that their information would remain confidential that there was no compulsion to participate and cooperate in the study and that they would not face any restrictions or problems if they decided not to participate.

## Method

The study sample consisted of 460 infertile couples who sought treatment at infertility treatment clinics (*Madar Sari Infertility Clinic* and *Kausar* Infertility *Clinic*) in the north of Iran. Sampling was performed randomly using a table of random numbers from the client list of the center after creating a case file for each couple and recording the case number. The method of sampling was systematic sampling. Accordingly, following the diagnosis of infertility and the couple’s referral to infertility treatment centers, the case numbers were randomly selected from the table of random numbers. Couples who met the inclusion criteria and expressed willingness to participate in the study were invited to complete the questionnaire. In addition, we we have adhered to appropriate reporting guidelines in the preparation of our research report. Specifically, STROBE (STrengthening the Reporting of OBservational studies in Epidemiology) for an observational study.

### Data analysis

Data analysis was conducted using SPSS version 22. The tests employed in the study included independent t-tests, Mann-Whitney tests, and chi-square tests. Simple logistic regression was used to assess the relationship between the main variables (categorized as below or above the threshold) and the independent variables. Furthermore, multiple logistic regression was employed to investigate the simultaneous effect of the independent variables on the main variables.

## Results

### Characteristics of participants

The mean age of the women participating in the study, the mean age of their husbands, the mean duration of marriage and regarding infertility, and the mean duration of infertility are reported in Table 1.

**Table 1:** Mean and standard deviation of quantitative variables of the research in Infertile Couples Referring to Infertility Treatment Clinics in Sari, 2020–2022

Variable	Mean (SD)
Woman’s age (year)	31.7 (5.71)
Man’s age (year)	35.22 (5.48)
Marriage duration (year)	6.86 (4.0)
Duration of infertility (year)	4.22 (3.11)
Duration of infertility treatment (year)	3.18 (2.63)

Demographic and clinical characteristics of infertile couples such as socioeconomic status, occupation, education level, etc. are available in Table 2.

**Table 2:** Demographic and clinical characteristics of Infertile Couples Referring to Infertility Treatment Clinics in Sari, 2020–2022

Characteristics	Women	Men	Couple
**Education level: N (%)**			
Primary and lower secondary	39 (8.5)	62 (13.5)	
Higher secondary and diploma	183 (39.8)	194 (42.1)	
Bachelor’s degree	191 (41.5)	147 (32)	
Master’s degree, Ph.D. and seminary education	47 (10. 2)	57 (12.4)	
**Occupation**:			
Housewife	353 (76.7)	-	
Employee	62 (13.5)	138 (30)	
Self-employed	-	272 (59.1)	
Hand worker	-	50 (10.9)	
Others	45 (9.8)	-	
**Place of residence**:			
Urban	-	-	344 (74.8)
Rural	-	-	116 (25.2)
**Economic status**:			
Unfavorable	-	-	45 (9.8)
Average	-	-	353 (76.7)
Favorable	-	-	62 (13.5)
**Ethnicity**:			
Persian	305(66.3)	303(65.9)	
Mazani	139(30.2)	143(31.1)	
Others	16(3.5)	14 (3)	
**Number of marriages**:			
1	417(90.7)	407(88.5)	
2 and more	43(9.3)	53(11.5)	
**Number of children from** **a previous marriage**:			
0	452(98.3)	435(93.6)	
1 and more	8(1.7)	25(5.4)	
**Cause of infertility**:			
Female factor	-	-	146(31.7)
Male factor	-	-	72(15.7)
Male and female factors	-	-	112(24.3)
Unknown	-	-	130(28.3)
**Number of previous treatments (IVF/ICSI)**:			
0	-	-	215(46.7)
1	-	-	178(38.7)
2	-	-	44(9.6)
3	-	-	12(2.6)
4 or 5	-	-	11(2.4)
**Type of infertility**:			
Primary	-	-	384(83.5)
Secondary	-	-	76(16.5)
**Willingness to continue treatment**:			
Yes	459(99.8)	458(99.6)	
No	1(0.2)	2(0.4)	
**Having an adopted child**:			
Yes	-	-	3(0.7)
No	-	-	457(99.3)
**History of depressive disorder**:			
Yes	31(6.7)	16(3.5)	
No	429(93.3)	444(96.5)	
**History of anxiety disorder**:			
Yes	68(14.8)	54(11.7)	
No	392(85.2)	406(88.3)	
**History of using psychiatric drugs**:			
Yes	32 (7)	21(4.6)	
No	428(93)	439(95.4)	
**History of suicide attempts**:			
Yes	4(0.9)	3(0.7)	
No	456(99.1)	457(99.3)	
**History of visiting a psychiatrist/psychologist**:			
Yes	42(9.1)	27(5.9)	
No	418(90.9)	433(94.1)	
**Systemic diseases such as diabetes and hypertension**:			
Yes	15(3.3)	23 (5)	
No	445(96.7)	437(95)	

**Table 3:** Determining and Comparing the Frequency of Emotional Risk Factors in Infertile Couples Referring to Infertility Treatment Clinics in Sari, 2020–2022

Emotional risk factors	Domain	Number of items	Threshold	Total (%)	Female	Male	*p*-value
Anxiety	-	5	Mild (< 24)	460 (50.00)	275 (59.8)	18.5 (40.2)	< 0.001
			Exposed to anxiety (≥ 24)	460 (50.00)	338 (73.5)	122 (26.5)	
Depression	-	7	Mild (< 4)	742 (80.7)	339 (73.7)	403 (87.6)	< 0.001
			Exposed to depression (≥ 4)	178 (19.3)	121 (26.3)	57 (12.4)	
Social support	-	5	Severe (> 15)	513 (55.8)	251 (54.6)	262 (57)	0.465
			Exposed to low level of social support (≤ 15)	407 (46.2)	209 (45.4)	198 (43)	
Understanding infertility problems	Acceptance	6	Severe (> 11)	665 (72.3)	320 (69.6)	345 (75)	0.066
			Exposed to acceptance (≤ 11)	115 (25)	140 (30.4)	115 (25)	
	Helplessness	6	Mild (< 14)	776 (84.3)	347 (81.3)	402 (87.4)	0.011
			Exposed to helplessness (≥ 14)	144 (15.7)	86 (18.7)	58 (12.6)	

Regarding emotional risk factors in infertile couples, there was a significant difference between women and men in terms of anxiety, depression, and helplessness and women were more exposed to these problems. Regarding the factors of social support and acceptance of fertility problems, there was no significant difference between women and men (Table 3).

After controlling other factors, the results of ordinal logistic regression and multivariable logistic regression revealed significant relationships between certain variables and the number of disorders in women. Specifically, the variables of history of depression disorder, history of anxiety disorder, SCREENIVF total score of spouse, and remarriage were significantly associated with the number of disorders in women. Women with a history of depression disorder had a 3.65 and 3.86 times higher chance of having a disorder compared to those without such a history. Similarly, women with a history of anxiety disorder had a 1.75 and 1.88 times higher chance of having a disorder compared to others. Furthermore, each additional disorder in men increased the chance of having an additional disorder in women by 2.35 and 2.67 times (Table 4).

**Table 4:** Factors related to SCREENIVF total score in women referring to infertility treatment clinics in Sari City in 2020–2022

Variable		Uni-variate analysis OR (95%CI)	*P*-value	Multi-variate analysis OR (95%CI)	*P*-value
Duration of marriage	-	0.97 (0.92–1.03)	0.310	0.08 (0.41–1.55)	0.506
Infertility treatment period	-	1.08 (0.99–1.18)	0.089	0.97(0.91–1.06)	0.092
Number of previous treatments	-	0.97 (0.77–1.23)	0.818	1.10 (0.89–1.36)	0.376
Total score of the spouse’s ScreenIVF	-	2.35 (1.99–2.77)	< 0.001	2.67(0.1–0.27)	< 0.001
Education	Primary and lower secondary	0.86 (0.36–2.07)	0.740	2.12(0.89–5.03)	0.522
	Higher secondary and diploma	1.25 (0.063–2.46)	0.525	1.56 (0.81–2.98)	0.183
	Bachelor’s degree	0.81 (0.43–1.53)	0.525	0.91(0.48–1.75)	0.781
	Master’s degree, Ph.D., and seminary education				
Occupation	Housewife	0.064 (0.35–1.15)	0.134	0.075 (0.42–1.2)	0.372
	Employee	0.71 (0.34–1.47)	0.71	0.78 (0.45–1.35)	0.799
	Self-employed and hand worker	-			
Place of residence	Urban	0.83(0.58–1.2)	0.389	0.92 (0.62–1.33)	0.255
	Rural				
Ethnicity	Persian	0.83 (0.58–1.2)	0.327	0.75 (0.27–2.06)	0.580
	Mazani and others				
Cause of infertility	Female factor	1.2(0.76–1.89)	0.428	1.29 (0.75–2.21)	0.355
	Male factor	1.14(0.67–1.95)	0.628	1.70(0.54–2.08)	0.855
	Female and male factors	1.37(0.85–2.21)	0.195	1.17(0.65–2.09)	0.600
	Unknown				
History of depressive disorder	Yes	3.65 (1.43–9.33)	0.007	3.86 (1.63–9.15)	0.002
	No				
History of anxiety disorder	Yes	1.75 (1.01–3.04)	0.045	1.88 (1.17–1.68)	0.025
	No				
Remarriage	Yes	0.39(0.16–0.99)	0.048	0.47(0.19–1.1)	0.004
	No				

After controlling for other factors, the logistic regression results indicated that age, spouse’s SCREENIVF total score, and education level were significantly related to men’s disorder, so each year of age was associated with a 7% lower chance of having more disorders in men. Men with a lower level of education had significantly higher odds of having more disorders than those with academic education. Additionally, each additional disorder in women increased the chance of having one more disorder in men by 94% (Table 5).

**Table 5:** Factors related to SCREENIVF total score in men visiting infertility treatment clinics of Sari in 2020–2022

Variable		Uni-variate analysis OR (95%CI)	*P*-value	Multi-variate analysis OR (95%CI)	*P*-value
Age	-	0.93 (0.98–2.54)	0.006	0.99 (0.95–1.50)	0.026
Spouse’s age	-	0.99 (1.04–2.69)	0.660	1.10(0.97–1.60)	0.577
Marriage duration	-	0.98 (1.05–2.66)	0.563	1.09 (1.20–1.16)	0.800
Infertility treatment period	-	1.08 (1.2–2.95)	0.132	1.12(1.20–1.21)	0.140
Number of previous treatments	-	1.14(1.45–3.13)	0.291	1.70(0.80–1.42)	0.799
Total score of the spouse’s ScreenIVF	-	1.94 (2.23–6.98)	< 0.001	2.35 (1.99–2.77)	< 0.001
Education	Primary and lower secondary	2.61 (6.09–13.66)	0.026	4.66(1.90-11.39)	0.001
	Higher secondary and diploma	2.01(3.94–7.43)	0.043	4.27(2.05–8.89)	< 0.001
	Bachelor’s degree	1.51(2.86–4.53)	0.205	1.73(2.94–4.86)	0.332
	Master’s degree, Ph.D., and seminary education	Reference			
Occupation	Employee	0.71(1.41–2.04)	0.330	1.62(0.57–4.54)	0.363
	Self-employed	0.97(1.68–2.65)	0.924	1.16(0.43–3.14)	0.773
	Hand worker				
Place of residence	Urban	1.26(2-3.54)	0.315	1.44(2.55–3.69)	0.421
	Rural				
Economic status	Unfavorable	1.77(3.98–5.86)	0.169	4.62(0.89–24.04)	0.073
	Average	1.64(2.89–5.13)	0.089	4.84(1.14–20.42)	0.094
	Favorable				
Ethnicity	Persian	0.94(1.37–2.56)	0.742	0.87(0.49–1.55)	0.645
	Mazani and others				
Number of children from previous pregnancies	0	0.31(1.36–1.37)	0.121	0.72(0.35–1.46)	0.361
	1≥				
Type of infertility	Primary	1.18(4.75–3.26)	0.812	0.48(0.20–1.18)	0.112
	Secondary				
History of depressive disorder	Yes	1.97(6.03–7.18)	0.233	0.44 (0.16–1.23)	0.119
	No				
History of anxiety disorder	Yes	1.75(3.47–5.75)	0.109	0.62(0.35–1.09)	0.094
	No				
History of taking psychiatric drugs	Yes	1.08(3.21–2.93)	0.896	0.45 (0.18–1.10)	0.085
	No				
History of seeing a psychologist/psychiatrist	Yes	2.34(6.53–10.43)	0.013	0.50(0.23–1.10)	0.045
	No				
History of underlying diseases	Yes	1.76(4.1–5.79)	0.193	0.57(0.54–1.31)	0.186
	No				
Type of infertility	Primary	1.18(4.75–3.26)	0.812	1.09(0.61–1.94)	0.772
	Secondary				
Cause of infertility	Female factor	0.7(1.12–2.02)	0.139	1.18(0.68–2.06)	0.555
	Male factor	0.95(1.67–2.58)	0.825	0.92(0.45–1.85)	0.808
	Female and male factor	1.13(1.86–3.11)	0.620	1.52(0.85–2.70)	0.156
	Unknown				

## Discussion

Most infertile couples acknowledge that infertility causes significant stress [8]. Infertility itself is a stress-inducing factor for couples, and the process of infertility treatment, with its uncertain outcomes, adds a dual physical and psychological burden for couples [9]. Even in cases where assisted reproductive technology (ART) results in successful pregnancy and vaginal delivery, women may still experience psychological distress. Furthermore, despite successful treatment, a history of infertility is associated with higher fertility-related anxiety, decreased postpartum self-confidence, parenting difficulties, and an increased risk of psychiatric disorders, including affective and psychotic disorders, within the first 90 days of postpartum [12]. Consequently, the present study aimed to investigate emotional risk factors and other related factors among infertile couples in Sari City in 2020–2022.

The study results revealed that half of the couples experienced anxiety. Furthermore, 19.3% of the couples reported depression, 44.2% perceived low social support, 27.7% had low acceptance of infertility problems, and 15.7% exhibited vulnerability. A cross-sectional study conducted by Prémusz et al. (2022) on infertile women reported that 56.9% experienced anxiety, 24.1% had depression, 37.79% had low social support, 20.7% had low acceptance, and 25.9% experienced feelings of helplessness [22].

Assisted reproductive methods are recognized as a complex and multidimensional source of stress and the treatment process is a significant stressor, and the unpredictable outcomes further contribute to this stress [23]. Infertility and the perceived lack of control during assisted reproductive treatments create a prolonged period of stress [24]. Unsuccessful treatment, in particular, can intensify distress, anxiety, and depression [25, 26]. while also diminishing the overall quality of life [18, 27]. In explaining this finding, previous studies suggest that women may be more accepting of reproductive methods. However, men may be more capable of accepting a life without children than women. The findings also indicate that women demonstrate a more significant commitment to continuing treatment. However, they are simultaneously more affected by their infertility problem and experience more emotional concerns compared to men. This disparity may help explain the differential impact of infertility diagnosis and assisted reproductive treatments on the psychological well-being of women and men [7]. Given that infertility and its treatment are significant sources of stress, this stress can trigger episodes of major depressive disorder, particularly in individuals with a history of depression [28]. A history of major depressive disorder serves as an important risk factor, predicting who will be vulnerable to episodes of major depression during treatment [29]. Overall, these studies highlight the interplay between anxiety and depression in infertile couples. Considering the results of the present study and previous research, it is crucial to screen women and their partners for a history of depression and anxiety disorder at the beginning of infertility treatment. Those identified as susceptible to these emotional risk factors can be provided with counseling resources and necessary psychological support.

The findings revealed that approximately 30% of the couples included in the study did not experience any emotional risk factors. Among those exposed to emotional risk factors, the highest percentage (31.8%) was attributed to one specific risk factor. Interestingly, only 2.6% of the couples simultaneously encountered five emotional risk factors. When comparing the frequency of SCREENIVF by gender, it was observed that the occurrence of SCREENIVF differed between men and women. Specifically, 31.3% of women were exposed to at least one risk factor, while 32.4% of men faced a similar situation. Around 25% of women and 35% of men did not encounter any emotional risk factors. Furthermore, the percentages of women and men exposed to all five risk factors were 4.3% and 0.9%, respectively. In a study by Verhaak et al. (2009), which aimed to assess emotional disorders in women undergoing IVF treatment, the SCREENIVF questionnaire identified 34% of women as being at risk of such disorders before commencing treatment. This percentage indicated that these patients exhibited clinical problems in at least one of the five emotional risk factors [14]. When examining the association between demographic-socio-clinical factors and anxiety, significant relationships were identified. Specifically, a history of depression disorder, anxiety disorder, and the spouse’s SCREENIVF score demonstrated a significant relationship with women’s anxiety. Additionally, the variables of education level, age, and the spouse’s SCREENIVF score showed a significant relationship with men’s anxiety.

The findings further indicated that the variables of history of depression disorder, history of anxiety disorder, and total SCREENIVF score of the spouse were significantly associated with a higher likelihood of experiencing more emotional risk factors in women. Thus, women with a history of depression and anxiety disorder had a greater chance of having multiple emotional risk factors. Moreover, each additional emotional risk factor in men increased the probability of having more emotional risk factors in women. Additionally, the total SCREENIVF score of the spouse and the level of education showed a significant relationship with the number of emotional risk factors in men. Thus, each unit of increase in the age of men decreased the likelihood of having more emotional risk factors in them. A possible explanation for this could be that various studies have shown that younger men are less likely to seek help from mental health professionals in times of mental problems [30, 31]. The chance of experiencing more emotional risk factors was significantly higher in men with a lower level of education compared to those with post-graduate education. This finding can be explained by the fact that an increase in the level of education is associated with a better understanding of multiple aspects of a situation and an increase in self-efficacy, which is a person’s belief in his ability to complete complex tasks and take responsibility [32].

Additionally, each additional emotional risk factor in women increased the likelihood of having one more emotional risk factor in men by 94%. Zhang et al. demonstrated in their study that anxiety or depression in each spouse functions as a risk factor for anxiety and depression in infertile couples [33]. One possible explanation for this matter is that a partner’s coping pattern influences the woman’s ability to cope with infertility and vice versa [34]. Similarly, Haimovici et al. found that men with anxious partners are more susceptible to experiencing symptoms of depression and anxiety [10]. Anxiety and depression are independent risk factors for long-term mental health problems [33]. One potential risk factor is a history of past episodes of major depression. Major depression is a recurrent disorder, and in the general population, it increases the likelihood of subsequent episodes [35].

The findings indicated that men with a lower level of education (ranging from elementary to diploma) had a higher likelihood of experiencing anxiety and depression than men with a post-graduate level of education or higher. This aligns with the results of Bjelland et al. (2008), demonstrating that higher education acts as a protective factor against anxiety and depression, while a lower level of education is significantly associated with these mental health conditions [36]. Several other studies have also identified the level of education as a risk factor for depression in the general population [37–39]. Individuals with a higher level of education tend to possess better coping strategies to deal with life’s stresses, leading to a more optimistic outlook and lower levels of depression and anxiety [39].

### Limitations

Our study also had limitations, mainly that it was conducted on people living in a single city, i.e., Sari, who is of Fars and Mazani ethnicity and could have differences with other ethnicities in different cities of Iran; therefore, we recommend conducting further studies in other cities and to compare the results with each other. Another limitation of our study was the cross-sectional design which made it difficult to derive causal interpretations of the relationships among variables.

## Conclusion

Recognizing the significance and impact of emotional risk factors on the outcomes of infertility treatment, it is indeed crucial to assess both husbands and wives for these factors. By conducting screenings and identifying emotional risk factors in infertile couples, the staff at infertility treatment centers can provide comprehensive and tailored care aimed at preventing the occurrence or worsening of these problems. This proactive approach not only addresses the emotional well-being of the individuals but also enhances the overall success and effectiveness of infertility treatment. By addressing emotional risk factors, healthcare providers can create a supportive and nurturing environment that contributes to the overall well-being of the couples throughout their fertility journey.

## Data Availability

The datasets used and/or analyzed during the current study are available from the corresponding author on reasonable request.

## References

[1] Mokhtari Sorkhani T, Alidousti K, Ahmadi A, Mirzaee M, Habibzadeh V, Namazian E, The effect of infertility counseling with couple therapy approach on the emotional level of infertile couples (RCT). Nurs Midwifery J. 2020;18(1):20–9.

[2] Gameiro S, Boivin J, Dancet E, de Klerk C, Emery M, Lewis-Jones C (2015). ESHRE guideline: routine psychosocial care in infertility and medically assisted reproduction-a guide for fertility staff. Hum Reprod.

[3] Kargar ST, Elyasi F, Jahanfar S, Peyvandi S, Aghmashhadi FYP, Hamzehgardeshi Z. Psychometric properties of the Persian version of the SCREENIVF among Persian infertile couples. 2022.

[4] Direkvand Moghadam A, Delpisheh A, Sayehmiri K (2013). The prevalence of infertility in Iran, a systematic review. Iran J Obstet Gynecol Infertility.

[5] Casu G, Gremigni P (2016). Screening for infertility-related stress at the time of initial infertility consultation: psychometric properties of a brief measure. J Adv Nurs.

[6] Bernd M, Schick M, Rösner S, Germeyer A, Strowitzki T, Moessner M (2020). Predictors for the early termination of a psychological intervention during treatment with assisted Reproductive technologies. Geburtshilfe Frauenheilkd.

[7] Molgora S, Baldini MP, Tamanza G, Somigliana E, Saita E (2020). Individual and Relational Well-being at the start of an ART treatment: a Focus on partners’ gender differences. Front Psychol.

[8] Crawford NM, Hoff HS, Mersereau JE (2017). Infertile women who screen positive for depression are less likely to initiate fertility treatments. Hum Reprod.

[9] Volmer L, Rösner S, Toth B, Strowitzki T, Wischmann T (2017). Infertile partners’ coping strategies are interrelated - implications for targeted psychological counseling. Geburtshilfe Frauenheilkd.

[10] Haimovici F, Anderson JL, Bates GW, Racowsky C, Ginsburg ES, Simovici D (2018). Stress, anxiety, and depression of both partners in infertile couples are associated with cytokine levels and adverse IVF outcome. Am J Reprod Immunol.

[11] Van Dongen AJ, Kremer JA, Van Sluisveld N, Verhaak CM, Nelen WL (2012). Feasibility of screening patients for emotional risk factors before in vitro fertilization in daily clinical practice: a process evaluation. Hum Reprod.

[12] Bhat A, Byatt N (2016). Infertility and perinatal loss: when the Bough breaks. Curr Psychiatry Rep.

[13] Huppelschoten AG, van Duijnhoven NT, Hermens RP, Verhaak C, Kremer JA, Nelen WL (2012). Improving patient-centeredness of fertility care using a multifaceted approach: study protocol for a randomized controlled trial. Trials.

[14] Verhaak CM, Lintsen AM, Evers AW, Braat DD (2010). Who is at risk of emotional problems and how do you know? Screening of women going for IVF treatment. Hum Reprod.

[15] Gana K, Jakubowska S (2016). Relationship between infertility-related stress and emotional distress and marital satisfaction. J Health Psychol.

[16] Huppelschoten AG, van Dongen AJ, Verhaak CM, Smeenk JM, Kremer JA, Nelen WL (2013). Differences in quality of life and emotional status between infertile women and their partners. Hum Reprod.

[17] Gameiro S, Boivin J, Domar A (2013). Optimal in vitro fertilization in 2020 should reduce treatment burden and enhance care delivery for patients and staff. Fertil Steril.

[18] Lopes V, Canavarro MC, Verhaak CM, Boivin J, Gameiro S (2014). Are patients at risk for psychological maladjustment during fertility treatment less willing to comply with treatment? Results from the Portuguese validation of the SCREENIVF. Hum Reprod.

[19] Irmak Vural P, Körpe G, Aslan E (2021). Validity and reliability of the Turkish version of Screening Tool on Distress in Fertility Treatment (SCREENIVF). Psychiatr Danub.

[20] Verhaak CM, Lintsen A, Evers A, Braat D (2010). Who is at risk of emotional problems and how do you know? Screening of women going for IVF treatment. Hum Reprod.

[21] Ghazanfarpour M, Sorkhani TM, Tajadiny L, Zeynivandnezhad F, Ahmadi A, Habibzadeh V (2023). Psychometric and clinical assessment of the Persian-SCREENIVF among infertile couples. Heliyon.

[22] Prémusz V, Ács P, Bódis J, Várnagy Á, Lászik Á, Makai A (2022). Introducing the Hungarian version of the SCREENIVF Tool into the clinical routine screening of emotional maladjustment. Int J Environ Res Public Health.

[23] Dunkel-Schetter C, Lobel M. Psychological reactions to infertility. Infertility: perspectives from stress and coping research. Springer; 1991. pp. 29–57.

[24] Chan CHY, Lau BHP, Tam MYJ, Ng EHY (2019). Preferred problem solving and decision-making role in fertility treatment among women following an unsuccessful in vitro fertilization cycle. BMC Womens Health.

[25] Gullo G, Cucinella G, Perino A, Gullo D, Segreto D, Laganà AS (2021). The gender gap in the diagnostic-therapeutic journey of the infertile couple. Int J Environ Res Public Health.

[26] Boivin J (2019). How does stress, depression and anxiety affect patients undergoing treatment?. Curr Opin Obstet Gynecol.

[27] Wdowiak A, Anusiewicz A, Bakalczuk G, Raczkiewicz D, Janczyk P, Makara-Studzińska M (2021). Assessment of quality of life in infertility treated women in Poland. Int J Environ Res Public Health.

[28] Nik Hazlina NH, Norhayati MN, Shaiful Bahari I, Nik Muhammad Arif NA (2022). Worldwide prevalence, risk factors and psychological impact of infertility among women: a systematic review and meta-analysis. BMJ Open.

[29] Holley SR, Pasch LA, Bleil ME, Gregorich S, Katz PK, Adler NE (2015). Prevalence and predictors of major depressive disorder for fertility treatment patients and their partners. Fertil Steril.

[30] Barney LJ, Griffiths KM, Jorm AF, Christensen H (2006). Stigma about depression and its impact on help-seeking intentions. Australian New Z J Psychiatry.

[31] Nam SK, Chu HJ, Lee MK, Lee JH, Kim N, Lee SM (2010). A meta-analysis of gender differences in attitudes toward seeking professional psychological help. J Am Coll Health.

[32] Saarni C. The development of emotional competence. Guilford Press; 1999.

[33] Zhang L, Shao H, Huo M, Chen J, Tao M, Liu Z (2022). Prevalence and associated risk factors for anxiety and depression in infertile couples of ART treatment: a cross-sectional study. BMC Psychiatry.

[34] Peterson BD, Newton CR, Rosen KH (2003). Examining congruence between partners’ perceived infertility-related stress and its relationship to marital adjustment and depression in infertile couples. Fam Process.

[35] Sim K, Lau WK, Sim J, Sum MY, Baldessarini RJ. Prevention of Relapse and recurrence in adults with major depressive disorder: systematic review and Meta-analyses of controlled trials. Int J Neuropsychopharmacol. 2015;19(2). 10.1093/ijnp/pyv076 PMC4772815 26152228

[36] Bjelland I, Krokstad S, Mykletun A, Dahl AA, Tell GS, Tambs K (2008). Does a higher educational level protect against anxiety and depression? The HUNT study. Soc Sci Med.

[37] Ansseau M, Fischler B, Dierick M, Albert A, Leyman S, Mignon A (2008). Socioeconomic correlates of generalized anxiety disorder and major depression in primary care: the GADIS II study (generalized anxiety and Depression Impact Survey II). Depress Anxiety.

[38] Barkow K, Maier W, Üstün TB, Gänsicke M, Wittchen H-U, Heun R (2003). Risk factors for depression at 12-month follow-up in adult primary health care patients with major depression: an international prospective study. J Affect Disord.

[39] Yang B, Zhang J, Qi Y, Wang P, Jiang R, Li H (2017). Assessment on occurrences of depression and anxiety and associated risk factors in the infertile Chinese men. Am J Men’s Health.

